# Pathophysiology of Cerebral Vascular Dysfunction in Pregnancy-Induced Hypertension

**DOI:** 10.1007/s11906-019-0961-8

**Published:** 2019-05-23

**Authors:** Subhi Talal Younes, Michael J. Ryan

**Affiliations:** 1 Department of Physiology & Biophysics, University of Mississippi Medical Center, 2500 North State Street, Jackson, MS 39216-4505 USA; 2 G.V. (Sonny) Montgomery Veterans Affairs Medical Center, Jackson, MS USA

**Keywords:** Preeclampsia, Cerebral circulation, Endothelial dysfunction, Blood–brain barrier, Myogenic response

## Abstract

**Purpose of Review:**

To review and summarize what is known about cerebrovascular derangements during preeclampsia.

**Recent Findings:**

Preeclampsia is a devastating disorder of pregnancy with no known cure. Little is known about the pathophysiological mechanisms which lead to the symptoms of the disorder, particularly with regard to individual vascular beds such as the cerebral circulation. Studies suggest that the cerebrovascular dysfunction characteristic of the preeclampsia syndrome is characterized by alterations in cerebral blood flow autoregulation and opening of the blood–brain barrier. Mechanistic studies demonstrate that the same circulating factors implicated in the pathophysiology of other vascular beds may be operative in the cerebral circulation as well. However, significant knowledge gaps still exist, highlighting the need for more intense research in this field.

**Summary:**

Little is known about cerebrovascular dysfunction during preeclampsia, and detailed mechanistic studies are needed to identify the molecular pathways involved, the interactions thereof, and how those pathways lead to clinical disease.

## Introduction

Preeclampsia is a multi-system disorder of pregnancy characterized by widespread vascular dysfunction. One of the most prominent organs to be affected is the brain. Indeed, central nervous system (CNS) manifestations such as headache, visual disturbances, alterations in consciousness, and seizures (eclampsia) are considered severe features of the disorder [1] and contribute significantly to maternal morbidity and mortality [2, 3]. Though significant insight into the pathophysiology of preeclampsia more broadly has been gained in recent years [4], the etiology of CNS dysfunction in this disorder is largely enigmatic. This paper briefly reviews pertinent clinical findings in this disorder and describes recent advances in basic science research that may offer mechanistic insights.

## Clinical Features of CNS Disease in Preeclampsia

Seizure was the first recognized feature of the obstetric disorder that would come to be recognized as eclampsia [5], underscoring the centrality of CNS dysfunction. Subsequent descriptions identified a set of prodromal symptoms, including hypertension, proteinuria, and headache. Thus, the term preeclampsia was coined. Therefore, in contrast to other named diseases, the term preeclampsia denotes a set of screening tests which identify those women who are at risk of developing eclampsia, rather than describing a defined pathology or pathophysiology [1]. This screening-based diagnosis is broadly recognized to confound most if not all clinical studies on preeclampsia by including many women who likely do not have the underlying disorder. This universal confounding likely explains why unambiguous descriptions of the pathophysiology of preeclampsia in humans are severely lacking, particularly with regard to the CNS. Nevertheless, the threshold for diagnosis is necessarily low, given the severity of the predicted outcome [6].

The syndrome of preeclampsia is believed to be caused in part by an ischemic placenta which releases factors into the maternal circulation that, when secreted in excess, lead to maternal vascular derangements. Specifically, inflammatory cytokines such as tumor necrosis factor-alpha (TNF-α) [7–9] and anti-angiogenic factors such as soluble Fms-like tyrosine kinase 1 (sFlt-1) have been implicated. sFlt-1 is a soluble splice variant of the vascular endothelial growth factor (VEGF) receptor containing only the ligand binding domain thereby acting as a decoy receptor effectively binding and sequestering free VEGF [10–12]. The mechanisms by which these factors lead to the maternal clinical syndrome remain largely unclear; however, recent work established endothelial dysfunction as a likely candidate. Several lines of evidence suggest endothelial dysfunction in preeclampsia. For example, endothelial cells isolated from preeclamptic pregnancies demonstrate stigmata of endothelial dysfunction in vitro [13–15], clinical studies report decreased in vivo endothelial function [16], and there is an increase in some, but not all, circulating markers of endothelial dysfunction in women with preeclampsia [17].

### Anatomic and Pathologic Description

In seeking to describe the pathophysiology of CNS disease in preeclampsia, it is important to consider the anatomic and physiologic data. It is helpful to conceive of the CNS manifestations of preeclampsia in three separate, albeit overlapping, categories: stroke, eclampsia (seizure), and preeclamptic encephalopathy. Stroke in preeclampsia is primarily related to severe hypertension with associated hemorrhage [18–20]. Interestingly, this is the sole rationale for lowering blood pressure in women with preeclampsia. Pharmacologic lowering of blood pressure is known to not impact the underlying disease course [6, 21].

With regard to eclampsia, autopsy examination demonstrates cortical edema and subcortical petechiae [22], with particular predilection for the occipital lobe. Histopathologically, these petechiae are revealed to represent perivascular microhemorrhage and are associated with hyperplastic arteriolosclerosis and fibrinoid necrosis of parenchymal vessels [22, 23]. Radiological imaging studies in women with eclampsia demonstrate white matter edema, again with particular predilection for the occipital lobe [22, 24]. Diffusion-weighted imaging revealed this edema to be largely vasogenic rather than cytotoxic in origin [24, 25], suggesting primary abnormalities of the cerebral vessels as the proximate cause.

Preeclamptic encephalopathy is a term used to describe the spectrum of neurologic symptoms that can occur in preeclampsia. These include headache, decreased level of consciousness, and visual disturbances, with the latter characterized by complete loss of vision, scotomata, blurred vision, diplopia, or photophobia. Radiologic imaging studies further demonstrate white matter edema with particular predilection for the occipital lobe. Taken together, the clinical and radiological findings in eclampsia and preeclampsia are reminiscent of a syndrome referred to as reversible posterior leukoencephalopathy syndrome (RPLS) [26, 27], also called posterior reversible encephalopathy syndrome (PRES).

Not all cases of preeclamptic encephalopathy are characterized by occipital edema. Rather, T2-hyperintense punctate lesions within the deep white matter are also described and may be more common than occipital edema depending on the cohort [22]. Such lesions have been described in patients with leukoaraiosis, or chronic white matter damage, most often in the periventricular region [28]. However, T2 hyperintense punctate lesions within the deep white matter (i.e., > 13 mm from the ventricular surface) are also observed and represent the early stages of disease. Histopathologic correlation reveals lipohyalinosis and occasional onion-skinning arteriolosclerosis [29], features commonly associated with chronic vascular disease. Contributing etiological factors include breakdown of the blood–brain barrier [30], chronic edema [31], and impaired blood flow autoregulation [32]. Risk factors for leukoaraiosis recapitulate the common cardiovascular disease risk factors—most notably hypertension, diabetes, smoking, and hypercholesterolemia [33–35].

### Physiologic Perturbances

Given the centrality of vascular dysfunction to other end-organ damage in preeclampsia, and the data implicating blood vessel abnormalities in the CNS, the cerebral circulation has become the chief focus of studies examining the pathophysiology of CNS dysfunction in preeclampsia. Functional data in pregnant women regarding the cerebral vasculature are sparse due to the necessity of many methods to utilize radioactive tracers or invasive methods that are contraindicated in pregnancy. Doppler-based ultrasound techniques have thus become the most commonly used method. This technique has limitations, as it can only measure flow velocity from which various parameters are then inferred [36–41].

With this in mind, most studies demonstrate indices of increased cerebral blood flow in women with preeclampsia and eclampsia as compared to gestational-age matched controls [42–44]. Congruent with these findings are recent studies demonstrating impaired cerebral blood flow autoregulation as determined by changes in cerebral blood flow velocity in the middle cerebral artery with spontaneous variations in mean arterial pressure [45].

A similar study utilizing comparable data collection techniques adopted a different analytical approach, regarding velocity data in terms of gain and phase [46]. With mean arterial pressure as the input and cerebral blood flow velocity as the output, a transfer function was constructed. Thus, gain represents the magnitude of change in cerebral blood flow velocity for a given change in mean arterial pressure and phase represents the time for measured cerebral blood flow velocity to adapt following a change in mean arterial pressure. This analysis reported that women with preeclampsia had an increased phase and a decreased gain. In contrast with the aforementioned studies, this suggests that the women with preeclampsia in this study had enhanced autoregulatory function, albeit with a higher latency for this autoregulation to take effect. In this same study, baseline cerebral blood flow velocity was indeed higher in the preeclamptic group consistent with the reports of others [42–45]. The reason for the disparity between these studies is not clear but may represent technical limitations in inferring blood flow from Doppler-measured velocity.

### Mechanisms

Regarding the molecular pathways that may play a role, a recent study by Ciampa, et al. [47••] provides interesting insight. In this study, the proteome was interrogated in cerebrospinal fluid obtained from women with preeclampsia and healthy controls, using an aptamer-based proteomic platform. Subsequent Ingenuity Pathway Analysis on differentially expressed proteins identified the TGF-beta, VEGF, angiotensin, and IL-6 pathways as the most prominent pathways affected. Given that all of these pathways are implicated in other facets of preeclamptic pathophysiology, this finding suggests that common themes of vascular dysfunction are operative throughout the body during preeclampsia. However, it is also possible that markers of CNS injury may be indicative of preeclampsia. For example, neurofilament, a component of the neuronal cytoskeleton and marker of neuroaxonal injury, performed similar to other emerging biomarkers in predicting later development of preeclampsia [48].

In summary, it remains difficult to draw definitive conclusions about the nature of cerebral pathophysiology during preeclampsia from current clinical studies. It appears that vasogenic edema represents a common pathology, but the proximate cause of this edema remains largely unknown. The mechanism likely involves vascular dysfunction of a similar nature to that seen in other vascular beds during preeclampsia.

## Basic Science Insights

Given the inherent limitations of obtaining physiological, cellular, and molecular data in pregnant women, basic science studies leveraging powerful animal models are clearly needed to shed light on the mechanisms underlying cerebrovascular dysfunction during preeclampsia. To our knowledge, there is only a single animal model that develops eclampsia [49••]. This is a transgenic mouse model that expresses the human allele ApoL1, an apolipoprotein implicated in chronic kidney disease [50]. During pregnancy, these mice develop seizures associated with significantly elevated systolic blood pressure (average > 140 mmHg), proteinuria, and elevated circulating sFlt-1 [49••]. Interestingly, this phenotype was present in wild-type female mice when mated with transgenic males, firmly implicating the fetal-placental unit as the underlying cause. The authors reported no gross pathologic abnormalities in the brains of these mice, noting only decreased staining for CD31, an endothelial cell marker, in the glomeruli of affected mice. It is unclear if similar perturbations of the endothelium were present in the cerebral vasculature. Thus, the precise mechanism of ApoL1 transgene-induced eclampsia in this model is unknown and warrants further study.

A recent genetic study identified ApoL1 risk alleles as being associated with incident preeclampsia [51•]. Interestingly, it was the fetal, not maternal, genotype that conferred the increased risk. Moreover, those women who developed preeclampsia in association with the fetal risk allele had an increased prevalence of cerebral symptoms as compared to women with preeclampsia without the associated fetal ApoL1 risk allele. In conjunction with the aforementioned animal study, these data raise tantalizing questions regarding the pathophysiology of ApoL1 risk variants and their effect on the CNS. Further studies examining this association are warranted.

Ryan, et al. [52] demonstrated that myogenic tone, which is crucial for normal cerebral blood flow autoregulation and protection of downstream delicate microvessels from barotrauma, is impaired in an experimental model that mimics preeclampsia by surgically reducing blood flow to fetal-placental units in rats. This decrease in myogenic tone was associated with decreased expression of Beta-ENaC, an important component of the vascular smooth muscle cell mechanosensor that mediates the vascular myogenic response [53]. A subsequent study confirmed the functional outcome of this mechanistic observation; namely, cerebral blood flow autoregulation is impaired during placental ischemia [54]. This latter study also demonstrated breakdown of the blood–brain barrier as evidenced by increases in brain water content and Evans blue dye extravasation. In contradistinction from studies on the aorta, there does not appear to be cerebral vascular endothelial dysfunction in middle cerebral arteries isolated from this model of placental ischemia [52]. This may be due to differences in methodologies assessing endothelial function or represent the fact that different pathogenic mechanisms are operative in different vascular beds. Whether endothelial dysfunction is present in the cerebral microvasculature, further downstream from these large conductance vessels is not known.

A commonly cited explanation for the development of preeclampsia is that an ischemic placenta elaborates numerous factors into the circulation which act on distant organs and cells to produce the clinical syndrome. Therefore, it is tempting to postulate that this same mechanism is operative in the cerebral circulation. Circulating factors act on the cerebral endothelium, pericyte, and vascular smooth muscle cell to induce the vascular changes described above. A study by Warrington, et al. [55] addressed this exact question. By infusing TNF-alpha into pregnant rats, many of the cerebral features of placental ischemia were recapitulated including increases in brain water content as a marker of cerebral edema. However, blood–brain barrier permeability as assessed by Evans blue dye extravasation was not altered by TNF-alpha infusion. In a companion study, blocking TNF-alpha with a soluble receptor attenuated placental-ischemia induced increases in blood–brain barrier permeability and brain water content. Moreover, unpublished data from our group indicate that TNF-alpha can act directly on vascular smooth muscle cells to downregulate Beta-ENaC, which presumably would perturb myogenic tone and lead to derangements in cerebral blood flow autoregulation.

Given its known role in other facets of preeclampsia, another contributing factor could be sFlt-1. This soluble receptor sequesters free VEGF, producing an anti-angiogenic imbalance in the maternal circulation during preeclampsia. Bean, et al. [56•] utilized a model of sFlt-1 and soluble endoglin (sEng, a soluble receptor for TGF-beta) infusion to interrogate the role of an anti-angiogenic balance in the cerebrovascular manifestations of preeclampsia. They found that this anti-angiogenic balance produced cerebral edema in areas of the brain supplied by the posterior circulation and impaired myogenic tone in the middle cerebral artery.

Collectively, data from experimental models suggest that many of the same factors which are operative in the peripheral circulation are active in the cerebral circulation and may directly cause the pathogenic perturbations seen in models of preeclampsia. Nevertheless, a unifying model which explains where (i.e., what cell type) and in what fashion (i.e., through what signaling pathways) these physiological derangements are accomplished by pathologic circulating factors is lacking and awaits further study.

## Conclusion

It is often difficult to integrate clinical and basic science data into a model of cerebrovascular dysfunction during preeclampsia. A current model which is widely recited in the literature is as follows. Placental ischemia, caused by impairments in spiral artery remodeling, preexisting maternal vascular disease, or other causes, leads to elaboration of vasoactive factors including TNF-alpha and sFlt-1. These factors act primarily on the endothelium but can act on other cell types, such as the vascular smooth muscle cell, to cause vascular dysfunction.

Loss of nitric oxide associated with vascular dysfunction causes a predisposition to cerebral vasospasm, perhaps underlying the ischemic lesions occasionally observed in preeclampsia. However, vasospasm and ischemia are not an inevitable consequence. Indeed, depending on the physiological milieu, endothelial dysfunction in the brain coupled with significant attenuation of vascular smooth muscle cell tone and the myogenic response can lead to hyperperfusion of the brain, particularly under conditions of systemic hypertension.

Thus, many cases of preeclampsia/eclampsia are complicated by a form of hypertensive encephalopathy (i.e., hyperperfusion injury). This model has been proposed previously, stretching back far in the literature [57]. However, several clinical observations also contradict this model. First, it is well known that eclampsia can occur at any blood pressure. Second, hydralazine is a direct acting vasodilator that is commonly used to decrease blood pressure in women with preeclampsia. A common side effect is headache, caused by dilation of the cerebral vasculature and increases in cerebral blood flow [58–61]. If cerebral hyperperfusion is operative in preeclampsia/eclampsia, hydralazine should markedly worsen the CNS symptoms of preeclampsia, and its widespread use should have resulted in an epidemic of eclampsia among these patients. However, this is not the case [62]. Thus, the underlying factors that contribute to CNS symptoms remain unclear, and the current literature suggests that multiple mechanisms are at play.

**Fig. 1:**
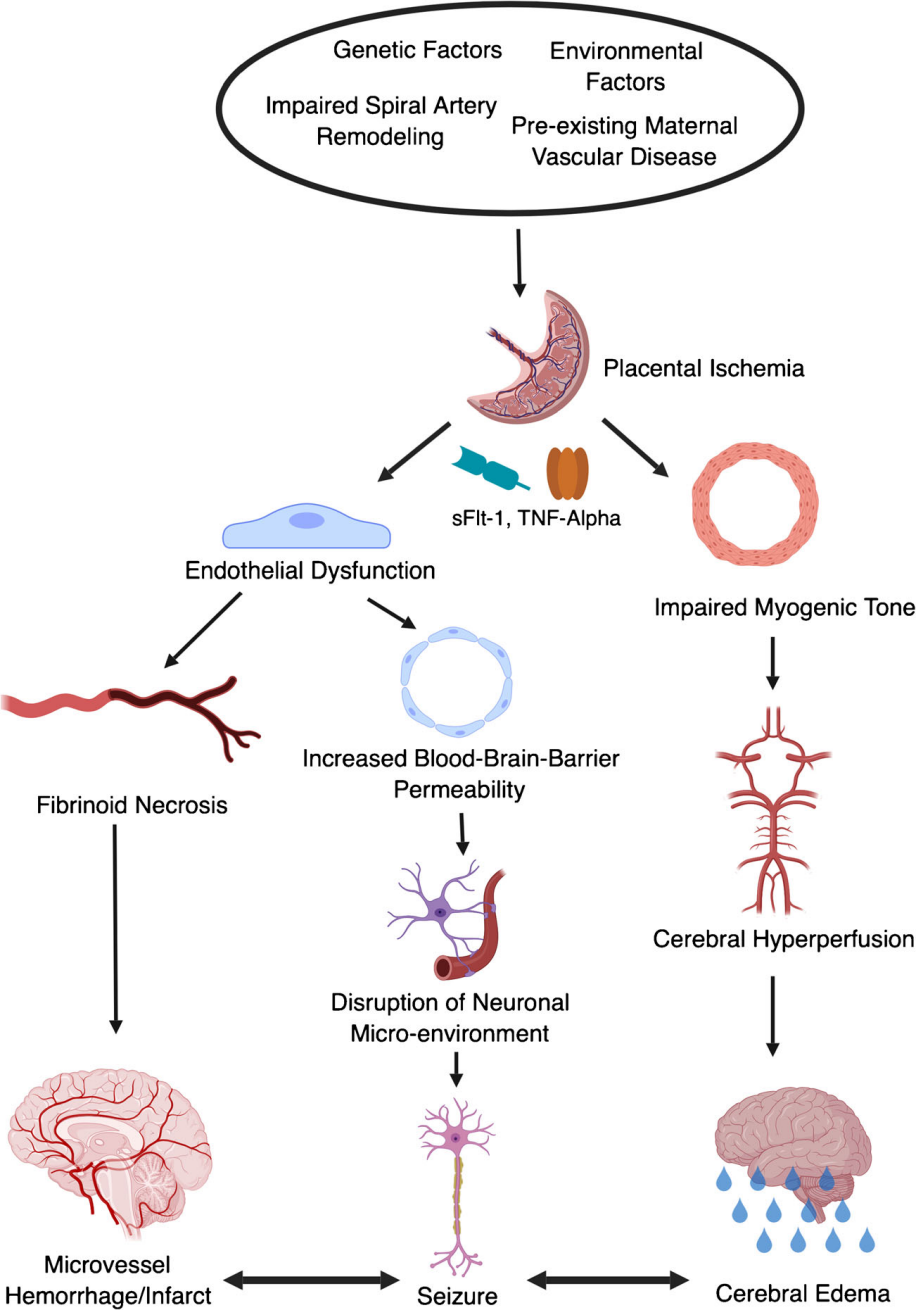
Proposed mechanism for cerebrovascular dysfunction during preeclampsia. Note the interactive nature of the three final pathologies, whereby infarction, seizure, and edema can causally overlap, each contributing to and worsening the others. sFlt-1, soluble fms-like tyrosine kinase 1; TNF-α, tumor necrosis factor-alpha; BBB, blood–brain barrier; CBF, cerebral blood flow

Based on studies from our laboratory and the literature, a modified mechanism might be considered (Fig. 1). Endothelial dysfunction directly leads to blood–brain barrier disruption and concomitant vasogenic edema, even at normal blood pressures. Hyperperfusion may be present, with particular predilection for the posterior circulation given the paucity of sympathetic innervation to this region. Thus, vasogenic edema is worsened, but is likely to occur even without hypertension. Edema and/or concomitant dysregulation of the delicate neuronal microenvironment (particularly electrolyte abnormalities) by blood–brain barrier breakdown leads to seizure. Due to endothelial cell activation, fibrinoid necrosis of the vessels may occur leading to bursting of delicate microvessels with microhemorrhages, particular with full blown seizure and concomitant neurovascular-coupling-mediated local vasodilation. Similarly, the absence of endothelial-derived vasodilatory factors may lead to vasospasm, resulting in cerebral ischemia. Thus, with endothelial dysfunction at its center, the full spectrum of cerebral complications of preeclampsia and eclampsia can be accounted for.
